# In Silico Repositioning of Cannabigerol as a Novel Inhibitor of the Enoyl Acyl Carrier Protein (ACP) Reductase (InhA)

**DOI:** 10.3390/molecules24142567

**Published:** 2019-07-15

**Authors:** Luca Pinzi, Christian Lherbet, Michel Baltas, Federica Pellati, Giulio Rastelli

**Affiliations:** 1 Department of Life Sciences, University of Modena and Reggio Emilia, Via Giuseppe Campi 103, 41125 Modena, Italy; 2 LSPCMIB, UMR-CNRS 5068, Université Paul Sabatier-Toulouse III, 118 route de Narbonne, 31062 Toulouse CEDEX 9, France

**Keywords:** drug repurposing, molecular modelling, cannabinoids, ligand-based virtual screening, docking, BEAR, natural products

## Abstract

Cannabigerol (CBG) and cannabichromene (CBC) are non-psychoactive cannabinoids that have raised increasing interest in recent years. These compounds exhibit good tolerability and low toxicity, representing promising candidates for drug repositioning. To identify novel potential therapeutic targets for CBG and CBC, an integrated ligand-based and structure-based study was performed. The results of the analysis led to the identification of CBG as a low micromolar inhibitor of the Enoyl acyl carrier protein (ACP) reductase (InhA) enzyme.

## 1. Introduction

In recent decades, increasing research efforts have been directed towards identifying novel drugs based on unexplored chemical scaffolds. However, the rate of drug approvals has become stable, with only a small number of the developed chemical entities entering in therapeutic use, or even in clinical trials [1,2,3,4]. As a consequence, discovery strategies have been adopted to reduce failures, time efforts and expenses; in this context, drug repurposing has become one of the most successful strategies to reduce failures typically associated with drug discovery. Drug repurposing consists of identifying novel therapeutic uses for already approved drugs and/or clinical candidates, as it might allow circumventing preclinical optimization issues, such as adverse toxicology profiles. Although most drug repurposing success stories derive from serendipity, current efforts are mainly directed toward rationally predicting repurposing through systematic analysis of bioactivity data with computational approaches. Indeed, in silico methods have been successfully used to help delineating new drug repurposing opportunities [5,6,7]. Several ligand-based and structure-based virtual screening approaches are currently available to support drug discovery programs. However, each in silico method alone could not be sufficiently able to model the complex interplay between drugs and targets, because of intrinsic limitations [8]. Therefore, the combination of ligand-based and structure-based methods is expected to: (i) provide more robust results; (ii) help overcoming intrinsic limitations of single approaches, and; (iii) complement each other in a drug discovery workflow [8,9,10]. Interestingly, the combination of ligand-based and structure-based approaches have already been successfully used to identify molecular targets for *Mycobacterium tuberculosis* phenotypic hits [11].

**Figure 1:**
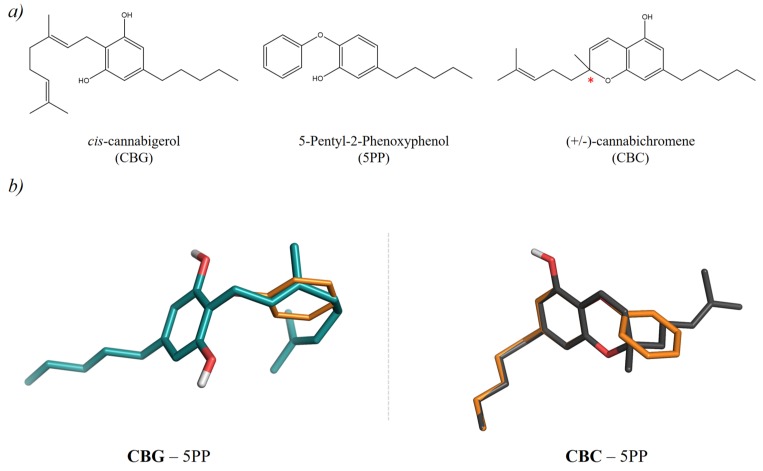
Chemical structures and ligand-based alignments of CBG and CBC predicted with ROCS [25]. Specifically, (**a**) reports the structures of CBG, 5PP and CBC. The chiral center in CBC is highlighted with a red star. (**b**) reports the shape-based alignment obtained for CBG (dark teal sticks) and the S stereoisomer of CBC (dark grey sticks) with the 5PP (orange thinner sticks) compound.

At present, most drug repurposing studies rely on the analysis of bioactivity data of compounds deriving from chemical synthesis. However, other valuable opportunities might come from natural products [12,13,14]. Natural products are characterized by a great structural diversity, and can provide novel chemical entities to be properly optimized in drug discovery campaigns. In this context, cannabinoids, which are terpenophenolics widely present in different varieties of *Cannabis sativa* L., are a very interesting class of bioactive compounds [15]. In particular, the pharmacological profile of non-psychoactive cannabinoids makes them the leading actors of the vast majority of scientific papers related to the fiber-type variety, which is commonly known as industrial hemp or hemp [16]. Among these compounds, cannabidiol (CBD) represents the best known example from a pharmaceutical point of view, possessing antioxidant, anti-inflammatory, antibacterial, anti-proliferative, neuroprotective and anticonvulsant properties [15,16]. Cannabigerol (CBG) and cannabichromene (CBC) are other non-psychoactive cannabinoids (Figure 1a), which can be found in *C. sativa* inflorescences; they are both characterized by antibacterial activity, together with anti-inflammatory and anti-proliferative properties [17].

Since the above-mentioned compounds represent good candidates for drug repositioning, the aim of this work was to develop and apply an integrated ligand- and structure-based in silico procedure to unveil possible biological targets of non-psychoactive cannabinoids to be used in future drug discovery campaigns.

## 2. Results and Discussion

To identify potential targets of CBG and CBC that could be of therapeutic interest, a 3D ligand-based virtual screening was first performed within the DrugBank database [18]. Indeed, this approach has already demonstrated to provide valuable results for drug repurposing, allowing the identification of structurally unrelated compounds with similar bioactivities [19]. Among the available databases, the DrugBank was selected because it provides a comprehensive list of approved and investigational drugs, with trustful bioactivity annotations on relevant therapeutic targets. The DrugBank was first prepared for the 3D ligand-based similarity analyses (see “Materials and Methods” section for details). Then, the DrugBank compounds were subjected to a 3D similarity screening against the generated CBG and CBC multi-conformer queries. This analysis allowed prioritizing potential therapeutic targets according to the degree of similarity of CBG and CBC with respect to the DrugBank compounds. Visual inspection of the predicted alignments allowed the identification of the Enoyl acyl carrier protein (ACP) reductase (InhA) enzyme as a potential target for both CBG and CBC. In fact, according to the ligand alignments, both cannabinoids resulted to be similar to 5-pentyl-2-phenoxyphenol (5PP, DrugBank ID: DB07178) (Figure 1b), which is a small molecular weight inhibitor of InhA [20]. Ligand similarities are reported in Table S1 of the Supporting Information. In particular, according to the obtained alignments, the 5-pentyl-1,3-dihydroxyphenyl moiety of CBG overlapped well with the 5-pentyl-2-phenoxy group of 5PP, while the 3,7-dimethylocta-2,6-dienyl moiety of CBG provides looser superimposition with the phenyl group of 5PP. Regarding CBC, the *n*-pentyl-chromene-5-ol group provided a less favorable overlap with the 5-pentyl-2-phenoxy group of 5PP, this moiety occupying significant larger volume with respect to the hydroxyphenyl group of 5PP.

Good ligand-based alignments of CBC and CBG with other cannabinoids reported in the DrugBank database were also found (see Table S1 in the Supporting Information), such as cannabidiol (DrugBank ID: DB09061) and dronabinol (DrugBank ID: DB00470), which have recently been approved for treating epilepsy in children and nausea associated with cancer chemotherapy, respectively [21,22]. However, we decided to focus our attention to the InhA enzyme, which is a validated target of well-known antitubercular drugs [23,24].

**Figure 2:**
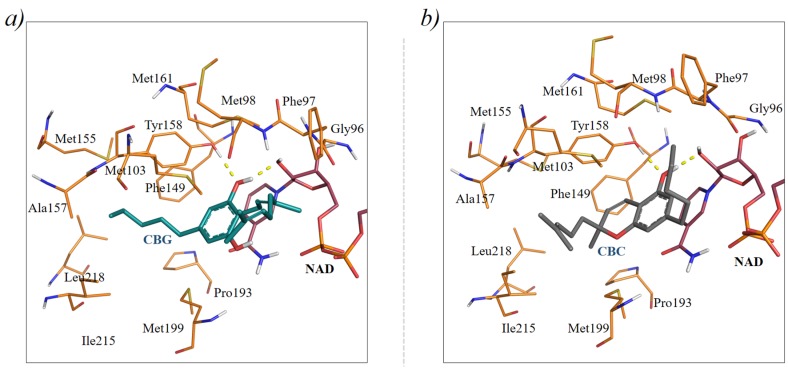
Docking poses of CBG and CBC into the 2B36 crystal structure predicted with Glide. Specifically, (**a**,**b**) report the predicted binding modes of CBG (dark teal sticks) and the S enantiomer of CBC (dark grey sticks) into the InhA receptor, respectively. NADH is reported as raspberry sticks.

Although CBC and CBG showed good shape similarity with the 5PP inhibitor, the two compounds were also docked in the InhA crystal structure (PDB code: 2B36) [20], as described in the “Material and Methods” section. This analysis made it possible to assess whether the investigated compounds also possess good steric and electrostatic complementarity with the InhA binding site. The 2B36 crystal structure was preferred among others available for InhA, because the enzyme is in complex with 5PP [20]. Indeed, the selection of suitable receptor conformations for docking by means of the similarity between the crystallographic and the screening ligands is among one of the most used methods to improve structure-based virtual screening results [9,26]. Docking analyses were performed with Glide and the Induced Fit Docking protocol available with the Schrödinger suite 2018-3, [27,28]. At this stage of the analysis, different docking protocols, i.e., rigid (Glide) and flexible (Induced Fit), were performed to evaluate whether small structural changes, due, e.g., to receptor flexibility of InhA, might affect ligand binding [29]. To assess the ability of the docking protocol to reproduce the native orientation of the crystallographic ligand, redocking of 5PP into its own 2B36 crystal structure was first performed as a control (see Figure S1 in the Supporting Information), with the evaluated Root Mean Square Deviation (RMSD) being below 2.0 Å. Then, docking of CBG and CBC was performed. A visual inspection of the docking complexes with the best score predicted with Glide showed that CBG could accommodate into the InhA binding site by adopting an orientation similar to that of 5PP (Figure 2a). Interestingly, one of the hydroxyl groups of CBG is involved in a H-bond network of interactions with both Tyr158 and the 2′-hydroxyl group of NADH, similarly to 5PP [20]. These interactions are recognized to be particularly important for the catalytic activity of InhA [20,30]. The phenol ring provides stacking interactions with the nicotinamide ring of NADH. The 3,7-dimethylocta-2,6-dienyl moiety was predicted to be accommodated between the Phe97, Met103 and the Ala198 residues, while the *n*-pentyl group binds near to the Phe149, Met155, Tyr158 and Leu218 residues, establishing hydrophobic contacts.

Different results were obtained for CBC, for which structure-based predictions did not agree with the ligand-based alignment. In particular, the S stereoisomer of CBC, which provided the best score in the docking calculations, was predicted to bind InhA with a binding mode that is head-to-tail with respect to that of 5PP in the crystal structure (Figure 2b and Figure S2). The different binding mode likely originates from steric repulsion of the 2-(4-methylpent-3-enyl) moiety of CBC with the NADH cofactor. In this binding mode, the S stereoisomer of CBC is engaged in H-bonds with both Tyr158 and the 2′-hydroxyl group of NADH, such as 5PP [20]. On the contrary, docking of the R enantiomer of CBC into the 2B36 crystal structure provided a binding pose that did not establish relevant H-bond interactions with the InhA binding site residues or NADH; therefore, this stereoisomer was not further considered. To further refine the results obtained with Glide, a rescoring of the predicted docking poses was performed with BEAR [31]. Indeed, BEAR has already demonstrated to improve docking results in a variety of virtual screening campaigns and retrospective validations, including also the enoyl ACP reductase target [32,33,34,35]. Therefore, it represents a valuable approach to refine docking results. Interestingly, BEAR provided MM-PBSA free-energies of binding clearly in favor of cannabigerol, the evaluated free energy scores being −28 Kcal·mol^−1^ and −20 Kcal·mol^−1^ for CBG and CBC (S stereoisomer), respectively.

Induced Fit docking experiments confirmed Glide results. In fact, docking poses with the best score predicted by the Induced Fit protocol superimposed well with those obtained by Glide, thus demonstrating that the predicted CBC and CBG binding modes were not affected by receptor flexibility of InhA (see Figure S3 of the Supporting Information).

Based on these results, CBG turned out to be the best candidate for inhibition of InhA. In fact, although CBC resulted structurally similar to the 5PP inhibitor according to the ligand-based analyses, it could not be accommodated as good as CBG within the InhA enzyme with docking. To test this prediction, CBG and CBC were purchased from Sigma-Aldrich (Milano, Italy) and then tested for the inhibition of the InhA enzyme activity, as described in the “Material and Methods” section [36]. Notably, the experiments confirmed that CBG inhibits InhA with low micromolar inhibitory activity, the evaluated IC_50_ being 5.2 ± 0.1 µM (see Table 1, and Figure S4 in the Supporting Information). On the contrary, CBC turned out to be inactive or scarcely active (Table 1), in agreement with the structure-based results.

Finally, docking complexes were used to suggest which structural modifications of CBG could potentially improve the activity of this cannabinoid. In particular, the substitution of the 3,7-dimethylocta-2,6-dienyl moiety of CBG with aromatic rings able to fit in the pocket lined by Met98, Phe97, Pro99, Gln100, Met103 and Ala198 could improve binding to InhA [20,37,38]. Likewise, substitutions of the *n*-pentyl moiety of CBG with cyclic aliphatic or aryl groups would provide additional van der Waals contacts with the Phe149, Met155, Pro193, Ile215 and Leu218 residues, which are expected to provide improved activity for InhA, as already observed for other InhA inhibitors [39].

## 3. Materials and Methods

### 3.1. Ligand-Based Virtual Screening on the DrugBank Database

Compounds with associated bioactivity annotations were first retrieved from the DrugBank database (www.drugbank.ca, accessed on April 20, 2018) [18]. Then, compounds with recognized toxic and reactive functional groups or transition metals, and a molecular weight lower than 150 or higher than 850 Da were removed, yielding a database of 6014 unique compounds. The FILTER software (OpenEye, Santa Fe, Mexico) was used for the filtering calculations [40]. Afterwards, all combinations of stereoisomers, ionization states and tautomers potentially present at neutral pH were generated for both the investigated cannabinoids and the filtered DrugBank ligands by using the Quacpac python toolkits [41]. Pre-defined chiralities were kept unaltered and pre-treated structures were minimized according to the MMFF force field [42]. Finally, up to 10 and 600 conformers were generated for the investigated cannabinoids and the filtered DrugBank compounds, respectively. A cutoff of 0.5 Å on RMSD and an energy window of 10 kcal/mol were used as parameters to accept conformers during the conformational sampling with the OMEGA software [40,43].

A multi-conformer versus multi-conformer 3D shape-based virtual screening was performed to evaluate the similarity profile of the investigated cannabinoids with respect to the filtered DrugBank compounds. The ROCS software (version 3.2.1.4) was used as the engine for the similarity calculations [25,44]. Finally, ligand alignments were visually inspected, and the activity annotations of the DrugBank compounds were carefully analyzed.

### 3.2. Structure-Based Virtual Screening on Enoyl Acyl Carrier Protein (ACP) Reductase

The InhA crystal structure (PDB code: 2B36) was first downloaded and pre-processed with the Protein Preparation Wizard module available within the Schrödinger Suite 2018-3 [20,45]. In particular, atom types and bond connectivity issues in the downloaded crystal structure were fixed. Moreover, missing side chains were rebuilt by using Prime (version 5.3) [46]. Then, hydrogen atoms were added to the pre-treated crystal structures and their coordinates were energy-minimized. Finally, ions, solvent and water molecules were removed.

Docking calculations were performed by using both Glide (version 8.0.012) and the Induced Fit Docking protocol available within the Schrödinger Suite (New York, NY, USA) 2018-3 [27,28]. The NADH cofactor was included in the docking calculations and considered as a part of the receptor. In the case of Glide, receptor grids were generated around the coordinates of the bound ligands (outer box of 10 Å × 10 Å × 10 Å), with rigid docking calculations performed by using default settings. Both the residues lining the protein-binding site and the NADH cofactor were considered as rigid elements in the Glide calculations, while the “flexible” sampling mode was used for the ligand to determine the optimal ligand conformation and orientation (default settings). For each ligand, only the pose with the best docking score was retained and visually inspected. In the case of Induced Fit Docking, default settings were used for receptor grid generation, while the calculations for docking the ligands into the 2B36 crystal structure were performed as follows. Firstly, the van der Waals radii of the protein and ligand were scaled by a factor of 0.8, afterwards the compounds were docked into the protein by using the default Glide SP protocol. Then, Prime was used to predict and optimize the protein side chains around the ligand. In this stage of refinement, the residues within 5 Å of each ligand pose were optimized, while the NADH cofactor was kept rigid. Other parameters were set to the default. Finally, the poses were re-docked by using the Glide XP protocol and then scored. Twenty poses with the best scores were retained for each ligand in the final step of the docking calculations to be visually inspected.

The docking procedure was validated by redocking the co-crystallized 5PP ligand into its crystal structure prior to the cannabigerol (CBG) and cannabichromene (CBC) screening. Afterwards, CBG and CBC were prepared with LigPrep for the docking calculations [47]. In particular, all combinations of stereoisomers, ionization states and tautomers potentially present at physiological pH in aqueous solution were first generated and then minimized according to the OPLS_2005 force field [48]. Finally, compounds were docked in the InhA active site. Moreover, the BEAR tool, which integrates molecular dynamics and binding free energy estimations with the aim of refining ligand-protein complexes and estimate binding energetics, was applied to further refine docking results obtained with Glide [31,32,33,34,35]. Default settings were used for rescoring the docking poses with BEAR [31]. A final step of visual inspection of the generated poses confirmed the selection of CBG and CBC as potential candidate inhibitors to be experimentally tested on InhA.

### 3.3. In Vitro Testing of the Compounds on the InhA Enzyme

CBG, CBC, NADH and the Triclosan standard control were purchased from Sigma-Aldrich. Stock solutions of the investigated compounds were prepared in DMSO to let their final concentration be equal to 5% *v/v*, in a final volume of 1 mL for all kinetic reactions. Kinetic assays were performed by using trans-2-dodecenoyl-coenzyme A (DDCoA) and wild type InhA, as previously described [49]. Briefly, reactions were performed at 25 °C in an aqueous buffer (30 mM PIPES and 150 mM NaCl pH: 6.8), containing additionally 250 µM cofactor (NADH), 50 µM substrate (DDCoA) and the tested compound (at 50 µM or 10 µM). Reactions were initiated by addition of InhA (50 nM final) and NADH oxidation was followed at 340 nm. The inhibitory activity of each derivative was expressed as the percentage inhibition of InhA activity (initial velocity of the reaction) with respect to the control reaction without inhibitor. Triclosan was used as the positive control. All activity assays were performed in triplicate. For the most potent compound, IC_50_ values were determined by using the 4-parameter curve-fitting software XLFit (IDBS), with at least six points.

## 4. Conclusions

In summary, we described the application of an integrated ligand-based and structure-based in silico repositioning approach to identify potential targets of non-psychoactive cannabinoids. The potential provided by the proposed approach was tested on cannabigerol (CBG) and cannabichromene (CBC), leading to the identification of CBG as a low micromolar inhibitor of the InhA enzyme. Indeed, naturally occurring cannabinoids are known to possess antibacterial activity in various bacterial strains [17,50] but, to the best of our knowledge, their biological target(s) have never been identified. Our study demonstrates that CBG is an InhA inhibitor. Interestingly, CBG is a small molecular weight compound with a good safety profile. Therefore, it represents a valuable starting point for the design of new synthetic derivatives with improved activity, thus paving the way to novel interesting possibilities for the treatment of infectious diseases. Finally, our study showed that integrating structure-based and ligand-based methods can lead to more accurate predictions of bioactive compounds [8]. This approach can be applied to efficiently repurpose any natural and synthetic ligand towards other therapeutic targets of interest.

## Figures and Tables

**Table 1:** Inhibitory activity of CBG and CBC. Triclosan was used as a positive control for the assays.

Compound	% Inhibition at 50 µM	IC_50_ (µM) ^a^
CBG	78	5.2 ± 0.1
CBC	31	^b^ nd
Triclosan (TCL)	100 (56% at 0.3 µM)	

**^a^** The reported IC_50_ values are the mean of three experiments ± SD. **^b^** Not determined.

## Supplementary Materials

The following are available online at https://www.mdpi.com/1420-3049/24/14/2567/s1. Figure S1: superimposition of the 5PP binding modes predicted by Glide and the Induced fit protocols with its crystallographic conformation; Figure S2: superimposition of the predicted ligand-based and structure-based (with Glide) poses of CBC; Figure S3: CBG and CBC binding modes predicted by the Induced Fit Docking protocol; Figure S4: the dose-response curve of CBG on InhA; Table S1: similarity values obtained with ROCS of CBC and CBG with respect to the DrugBank ligands.
