# Supplementary material for: In Silico Repositioning of Cannabigerol as a Novel Inhibitor of the Enoyl Acyl Carrier Protein (ACP) Reductase (InhA)
Source: Molecules. 2019 Jul 15;24(14):2567. doi: 10.3390/molecules24142567 (PMC6680637; doi:10.3390/molecules24142567)
Supplement: Supplementary file 1 [file molecules-24-02567-s001.pdf]

In silico repositioning of cannabigerol as a novel inhibitor of the Enoyl acyl carrier protein (ACP) reductase (InhA) protein

Luca Pinzi [1], Christian Lherbet [2], Michel Baltas [2], Federica Pellati [1], Giulio Rastelli [1,*]

[1] Department of Life Sciences, University of Modena and Reggio Emilia, Via Giuseppe Campi 103, 41125 Modena, Italy.

[2] LSPCMIB, UMR-CNRS 5068, Université Paul Sabatier-Toulouse III, 118 route de Narbonne, 31062 Toulouse Cedex 9, France.

* Correspondence: giulio.rastelli@unimore.it; Tel.: +39-059-2058564 (G.R.)

TABLE OF CONTENTS

Figures

Figure S1…………………. …..………………………………………………..…………… 3

Figure S2………………………………...……………………………….…………… 4

Figure S3……………………………………...………………………….…………… 5

Figure S4……………………………………...………………………….…………… 6

Tables

Table S1……………………………………………………………………...………7

Figure S1: Superimposition of the 5PP binding modes predicted by Glide and by the *Induced Fit* protocols with its crystallographic conformation. In particular, ***panels a)*** and ***b)*** report the superimposition of the 5PP binding modes predicted with Glide and the *Induced Fit* protocol to the crystallographic conformation of the same ligand, respectively. As shown, the predicted 5PP poses provided RMSDs of 1.1 Å (Glide) and 0.5 Å (*Induced Fit Protocol*) with respect to the experimentally derived conformation of the same ligand. The crystallographic conformation of 5PP is represented as orange thinner sticks, whereas the predicted binding poses are represented as blue sticks.

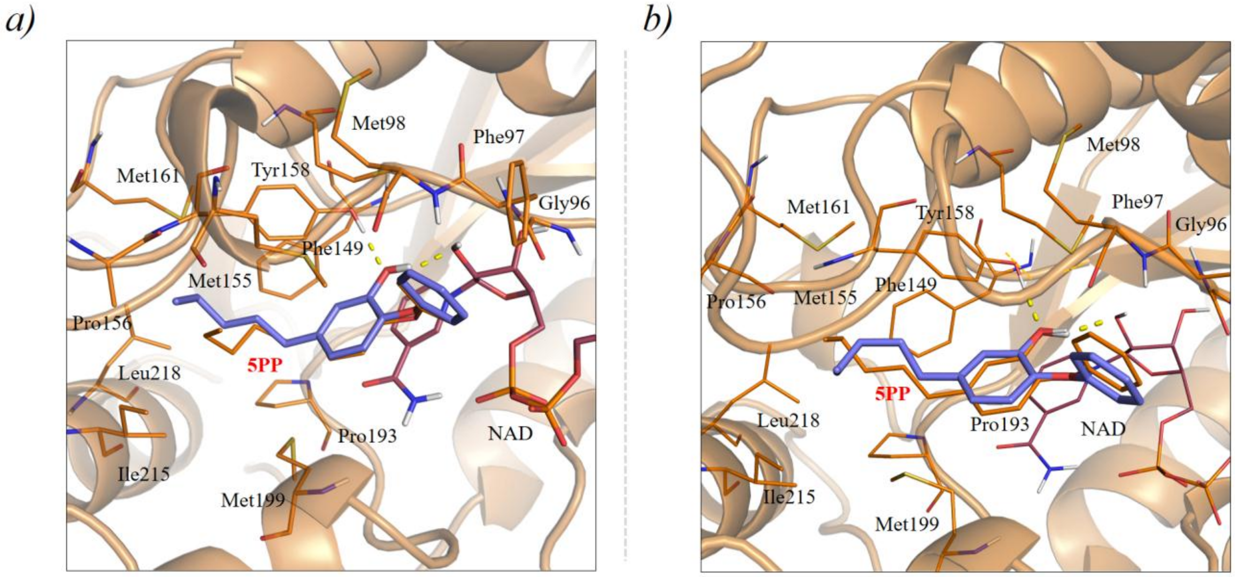

Figure S2: Superimposition of the ligand-based and structure-based poses predicted for CBC, and common sub-structure with 5PP. In particular, ***panel a)*** reports the superimposition of the ligand-based and structure-based poses of CBC predicted with ROCS and Glide, respectively. The conformation of CBC obtained by means of the shape-based screening with ROCS is represented as light grey sticks. The crystallographic conformation of 5PP is represented as orange thinner sticks. The docking pose of CBC into the 2B36 receptor predicted by Glide is represented in dark grey sticks. As shown, the two CBC poses are head to tail (RMSD of 6.3Å). ***Panel b)*** reports the chemical structures of CBC and 5PP, with highlighted their common sub-structure.

a)

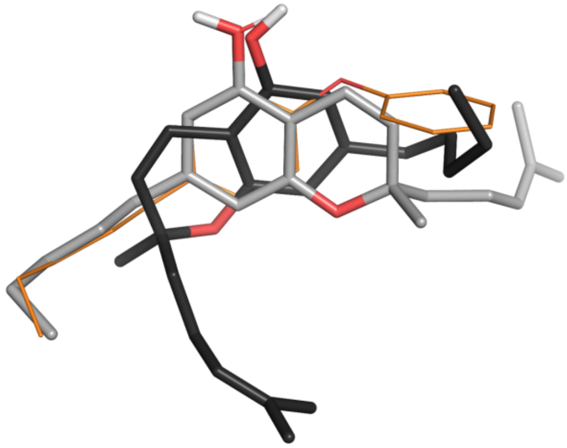

b)

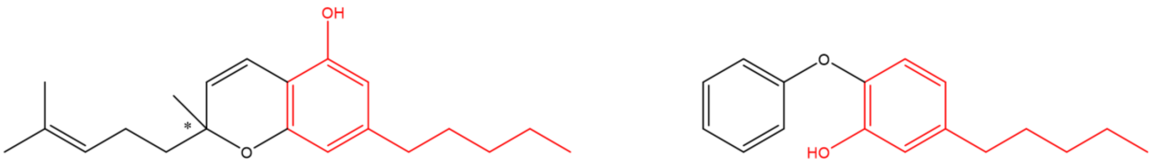

CBC **5PP**

Figure S3: Representative *Induced Fit Docking* poses of CBG and CBC into the 2B36 crystal structure. ***Panels a)*** and ***b)*** report the predicted binding modes of CBG (dark teal sticks) and CBC (dark grey sticks) into the InhA enzyme, respectively. The NADH cofactor is represented as raspberry sticks. The image was created with PyMol (The PyMOL Molecular Graphics System, Version 1.8, Schrödinger, LLC).

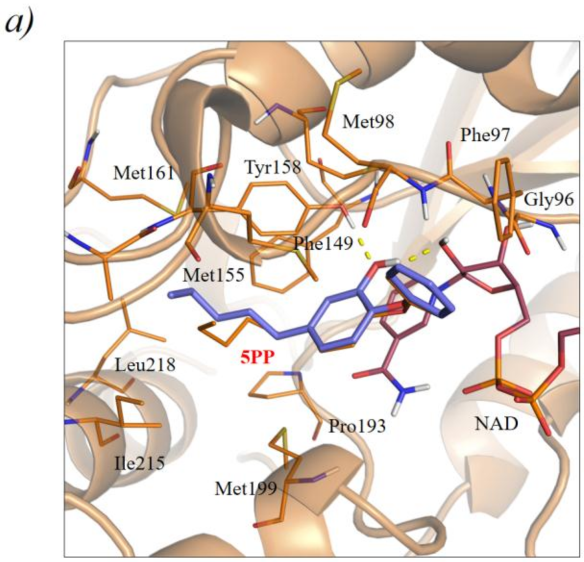

Figure S4: **Dose-response curve of CBG for the inhibition of the InhA enzyme.**

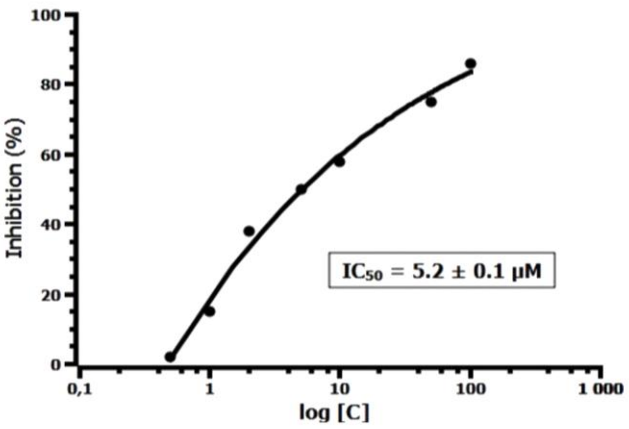

Table S1: Top five similarity values identified for CBG and CBC with respect to the ligands reported in the DrugBank database. The similarity estimations were performed through a multi-conformer *versus* multi-conformer 3D shape-based screening. Therefore, the reported similarity scores are related to the query and screening compound conformers that provided the best shape-based alignment. DrugBank compounds that resulted similar with both CBG and CBC are shown in bold. Similarity records identified for the 5PP ligand (DrugBank ID: DB07178) are highlighted in red.

Query	*Compound ID*	*Tanimoto Combo*†	*Shape Tanimoto*	*Color Tanimoto*	*Name*	*Groups*	*Targets*
**CBG**	**DB09061**	**1.307**	**0.671**	**0.636**	**Cannabidiol**	**Approved, Investigational**	**Cannabinoid receptor 1; Cannabinoid receptor 2***
	**DB07178**	**1.197**	**0.78**	**0.416**	**5-Pentyl-2-Phenoxyphenol**	**Experimental**	**Enoyl-[acyl-carrier-protein] reductase [NADH]**
	DB08690	1.174	0.818	0.357	Ubiquinone-2	Experimental	Succinate dehydrogenase flavoprotein subunit; Succinate dehydrogenase iron-sulfur subunit*
	**DB00470**	**1.093**	**0.633**	**0.459**	**Dronabinol**	**Approved, Illicit**	**Cannabinoid receptor 1; Cannabinoid receptor 2**
	DB03152	1.087	0.747	0.34	B-2-Octylglucoside	Experimental	DNA polymerase I, thermostable; Aquaporin Z*
**CBC**	**DB00470**	**1.386**	**0.689**	**0.697**	**Dronabinol**	**Approved, Illicit**	**Cannabinoid receptor 1; Cannabinoid receptor 2**
	**DB09061**	**1.228**	**0.62**	**0.608**	**Cannabidiol**	**Approved, Investigational**	**Cannabinoid receptor 1; Cannabinoid receptor 2***
	DB07918	1.214	0.691	0.522	2-Heptyl-4-Hydroxy Quinoline N-Oxide	Experimental	Fumarate reductase flavoprotein subunit; Fumarate reductase iron-sulfur subunit*
	**DB07178**	**1.193**	**0.694**	**0.499**	**5-Pentyl-2-Phenoxyphenol**	**Experimental**	**Enoyl-[acyl-carrier-protein] reductase [NADH]**
	DB00486	1.168	0.647	0.521	Nabilone	Approved, Investigational	Cannabinoid receptor 1; Cannabinoid receptor 2

* The compound showed activity annotations on more than two targets.

† Tanimoto Combo scores span from 0.0 to 2.0, as they result from the sum of shape fitting (Shape Tanimoto) and atom type superimposition (Color Tanimoto) similarity scores. The higher is the Tanimoto Combo score evaluated between two ligands, the higher are their shape similarity and atom type superimposition.
